# Function of YY1 in Long-Distance DNA Interactions

**DOI:** 10.3389/fimmu.2014.00045

**Published:** 2014-02-10

**Authors:** Michael L. Atchison

**Affiliations:** ^1^Department of Animal Biology, School of Veterinary Medicine, University of Pennsylvania, Philadelphia, PA, USA

**Keywords:** YY1, polycomb, condensin, cohesin, DNA loops, immunoglobulin loci

## Abstract

During B cell development, long-distance DNA interactions are needed for V(D)J somatic rearrangement of the immunoglobulin (Ig) loci to produce functional Ig genes, and for class switch recombination (CSR) needed for antibody maturation. The tissue-specificity and developmental timing of these mechanisms is a subject of active investigation. A small number of factors are implicated in controlling Ig locus long-distance interactions including Pax5, Yin Yang 1 (YY1), EZH2, IKAROS, CTCF, cohesin, and condensin proteins. Here we will focus on the role of YY1 in controlling these mechanisms. YY1 is a multifunctional transcription factor involved in transcriptional activation and repression, X chromosome inactivation, Polycomb Group (PcG) protein DNA recruitment, and recruitment of proteins required for epigenetic modifications (acetylation, deacetylation, methylation, ubiquitination, sumoylation, etc.). YY1 conditional knock-out indicated that YY1 is required for B cell development, at least in part, by controlling long-distance DNA interactions at the immunoglobulin heavy chain and Igκ loci. Our recent data show that YY1 is also required for CSR. The mechanisms implicated in YY1 control of long-distance DNA interactions include controlling non-coding antisense RNA transcripts, recruitment of PcG proteins to DNA, and interaction with complexes involved in long-distance DNA interactions including the cohesin and condensin complexes. Though common rearrangement mechanisms operate at all Ig loci, their distinct temporal activation along with the ubiquitous nature of YY1 poses challenges for determining the specific mechanisms of YY1 function in these processes, and their regulation at the tissue-specific and B cell stage-specific level. The large numbers of post-translational modifications that control YY1 functions are possible candidates for regulation.

## The Early Days

Yin Yang 1 (YY1) was first identified in 1985 as a factor that yielded an *in vivo* B cell-specific DMS methylation interference pattern over the immunoglobulin heavy chain (IgH) intron enhancer (1, 2). The enhancer site that bound YY1 was defined as the μE1 site (3) and nuclear factors that bound to this sequence were identified by EMSA (4). Our laboratory isolated a cDNA clone expressing a protein that bound to the Igκ3′ enhancer as well as the IgH μE1 site and named the protein NF-E1 (5). Simultaneously the factor was cloned by Tom Shenk’s laboratory and named YY1 (6) based on its ability to bind the adenoviral P1 promoter and both activate and repress transcription, by Robert Perry’s laboratory and named delta (7) due to its binding to the delta motif in the promoters of ribosomal protein genes, and by Keiko Ozato’s laboratory and named UCRBP based on its ability to bind to the upstream control region of retroviral LTRs (8). Ultimately, the name YY1 was adopted by all.

**Figure 1:**
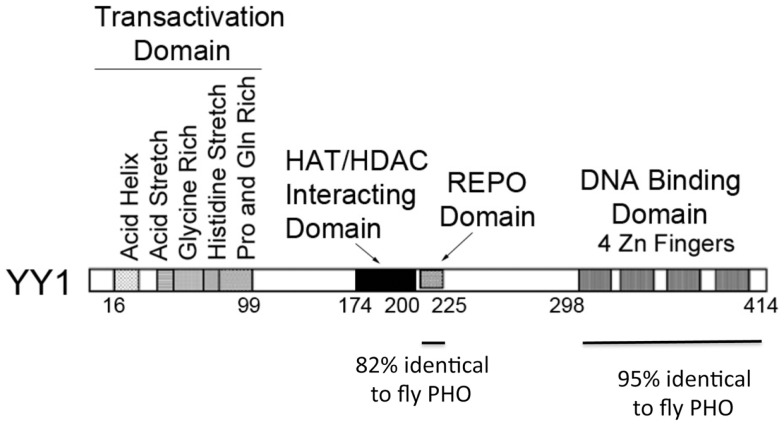
**Diagram of YY1 domains and functions**. Domains of YY1 are indicated with specific functions listed. The regions of similarity to *Drosophila* Plieohomeotic are indicated below the diagram.

Yin Yang 1 contains four zinc fingers at its carboxyl terminus (amino acids 298–414) and a region rich in alanine and glycine between amino acids 154 and 201. The first 100 amino acids of YY1 encode several notable features. Sequences 43–53 contain 11 consecutive acidic residues while amino acids 70–80 consist of 11 consecutive histidine residues. These two segments are separated by a region rich in glycine (residues 54–69). In addition, sequences 16–29 have the potential to form an amphipathic negatively charged helix and sequences 80–100 are rich in proline and glutamine. Sequences near the carboxyl terminus (333–397), which overlap the YY1 zinc fingers, and sequences 170–200 have been reported to be involved in transcriptional repression (6, 9–15). These sequences are known to physically interact with a variety of transcriptionally important proteins including TBP, p300, c-myc, and HDAC2 (16). YY1 sequences important for transcriptional activation reside near the amino-terminus (9, 12, 13, 17). Figure 1 shows various sequence features and functional domains of YY1.

## Diverse and Complex Roles of YY1

Over the past 22 years, multiple diverse YY1 functions have been identified. YY1 is crucial for embryonic development because homozygous mutation of the *yy1* gene in mice results in peri-implantation lethality (18). YY1 is implicated in lineage differentiation of skeletal and cardiac muscle, and in cell growth control (13, 17, 19–24), as well as disease pathways such as dystrophic muscle disease (25–27). YY1 and its target genes are also believed to be central regulators of germinal center B cell development (28), and YY1 has been suggested to regulate genomic targeting of activation induced cytidine deaminase (AID) (29). YY1 is implicated in a number of cancers (30–32), and is overexpressed in B cell lymphomas that depend on AID function. YY1 is associated with B cell transformation and tumor progression in diffuse large B cell lymphoma (DLBCL) (33, 34), and high levels of YY1 expression are associated with reduced patient survival in DLBCL as well as follicular lymphoma. CTCF–YY1 elements are clustered in the imprinting domain of Tsix (35) and YY1 docks Xist particles on the X chromosome via DNA and RNA interactions during X chromosome inactivation (36). YY1 can also control imprinting at the Peg3 and Gnas domains (37). YY1 can control human immunodeficiency virus (HIV) gene expression and viral titers, and deletion of YY1 binding sites in regulatory regions of human papilloma viruses correlates with increased viral gene expression and the development of cervical cancer (38–46). Thus, YY1 function is related to transcriptional regulation, embryonic development, X-chromosome inactivation, imprinting, oncogenesis, viral gene expression, epigenetic function, and a growing list of diseases.

## Identification of the PcG Function of YY1

A significant new function of YY1 was suggested in 1998 when the Kassis laboratory cloned the *Drosophila* Pleiohomeotic (PHO) sequence and observed similarity to YY1 (47) (Figure 1). Girton and Jeon (48) demonstrated that PHO is a Polycomb Group (PcG) protein, a family of proteins involved in epigenetic chromosomal condensation, stable transcriptional repression, control of cell proliferation, hematopoietic development, as well as stem cell self-renewal. This raised the exciting possibility that YY1 is a vertebrate PcG protein. PHO is highly homologous to YY1 in two regions. These two regions include YY1 sequences 296–414 and 205–226 (the corresponding segments in PHO are residues 357–475 and 148–169, respectively). Sequences 298–414 constitute the four YY1 zinc fingers. The homology over this region is extraordinary for organisms as diverse as flies and humans (112 identities out of 118; 95%). Within this segment, zinc fingers 2 and 3 are 100% identical. The 205–226 segment is also highly homologous (18/22; 82% identity). Outside of these regions of high similarity, YY1 and PHO showed no discernible similarity. PHO does not contain an obvious transcriptional activation domain and lacks YY1 structural features such as acid and histidine stretches. However, the two regions of high similarity between YY1 and PHO, and their similar spatial locations within the proteins, suggested that they might carry out some of the same functions in vertebrates and flies, respectively.

Prompted by the possibility that YY1 functions as a PcG protein, we tested this hypothesis using a *Drosophila in vivo* transcription system, as well as a phenotypic correction assay. Our results showed that human YY1 does indeed function as a PcG protein *in vivo* (49–51). We found that YY1 can repress transcription in a PcG-dependent fashion, can phenotypically correct *pho* mutant flies, and can recruit PcG proteins to specific DNA sequences resulting in tri-methylation of H3 lysine 27 (49–51). The mechanisms responsible for targeting mammalian PcG proteins to specific DNA regions has long been proven enigmatic because none of the components of the PcG complexes bind to specific DNA sequences, yet the PcG complexes associate with specific DNA regions *in vivo*. Our demonstration that YY1 is a mammalian PcG protein with high affinity sequence-specific DNA binding activity suggested that YY1 is a crucial factor for targeting specific proteins to specific DNA sequences. The role of YY1 in PcG targeting has been confirmed in a number of studies (52–55) though clearly other factors are involved as YY1 (and PHO) does not co-localize with PcG proteins in all cell types (56–58). A particularly exciting aspect of YY1 PcG function is that PcG proteins are known to contribute to B cell development, and the PcG protein EZH2, like YY1, is required for Ig locus contraction (further explained below) (59). Nucleation of PcG proteins to specific target DNA sites by YY1 within the Ig loci thus opens up a new avenue for mechanistic evaluation of B cell development and Ig locus contraction, because PcG proteins are capable of mediating long-distance DNA interactions (60).

## The YY1 REPO Domain

Using a fly transgenic approach, we set out to identify the YY1 sequences involved in PcG function (61). We found that the region of 82% YY1-PHO identity (the 25 amino acids between residues 201 and 226), when fused to a heterologous GAL4 DNA binding domain, was necessary and sufficient for PcG-dependent transcriptional repression. Amazingly, this small 25 amino acid segment was also necessary and sufficient for recruitment of PcG proteins to DNA resulting in tri-methylation of H3 lysine 27. Therefore, we named YY1 sequences 201–226 the REPO domain for their ability to REcruit Polycomb (61). A REPO domain YY1 mutant (Δ201–226) can mediate nearly all YY1 functions such as DNA binding, transcriptional activation, transient transcriptional repression, and interaction with HDAC proteins. However, this mutant fails to carry out YY1 PcG functions and fails to recruit PcG proteins to DNA (61). How the YY1 REPO domain recruits PcG proteins to DNA is now being elucidated. Two homologous proteins, YAF2 and RYBP, were previously identified as YY1 interacting proteins (62, 63). Functionally, RYBP associates with a subset of PcG complexes named PRC1L4 (64) and is involved in the repressive function of *hoxD11.12*, a mammalian “PRE-like” sequence (65). YAF2 was first identified by its ability to bind to YY1 (63) and we found YAF2 can interact with the REPO domain perhaps functioning as a bridge protein in PcG recruitment (52, 66). The importance of the YY1 REPO domain for B cell development is discussed below.

## Structure of Immunoglobulin Loci during B Cell Development

B cell development involves progression from Lin^−^Sca-1^+^c-kit^+^ (LSK) progenitor cells through a number of intermediate B cell stages including pro-B, pre-B, immature B, mature B, and plasma cell stages. The early stages of B cell development can be delineated by the rearrangement status of the immunoglobulin heavy and light chain genes. Both heavy and light chain genes are produced during early, antigen-independent B cell development by a somatic rearrangement process that links together either V, D, and J segments (heavy chain), or V and J segments (light chain) to produce functional Ig genes (67–70). The Ig loci are huge (2.4–3.2 Mb) and for rearrangement of distal variable region genes to occur, the loci must go through a physical contraction process. Prior to the onset of rearrangement, Ig loci reside at the nuclear periphery in an “extended” configuration. However, at the pro-B cell stage, when the heavy chain genes undergo rearrangement, the loci take up an intranuclear localization with concomitant contraction of the loci (heavy chain first followed by light chain) (71–74). While IgH DJ and proximal V_H_ to D and Vκ to Jκ rearrangements can occur without contraction, the distal V genes require locus contraction and looping for rearrangement (71–73, 75–77).

**Figure 2:**
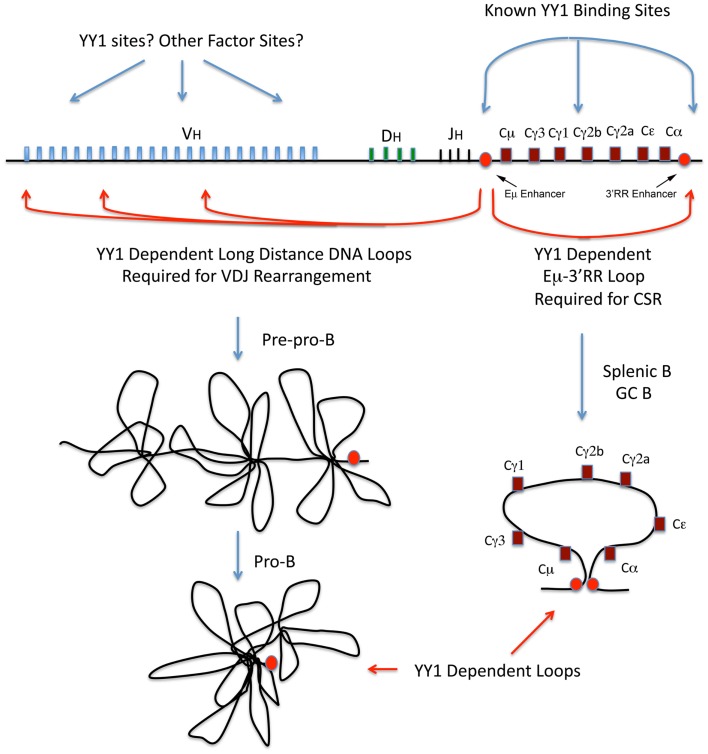
**Immunoglobulin heavy chain locus diagram with V, D, J, and C regions indicated and locations of known YY1 binding sites, and the approximate positions of long-distance DNA loops that are YY1-dependent**. The red circles represent the IgH intron and 3′RR enhancers. The left panel models rosette-like loops encompassing the V_H_ regions at the pre–pro-B and pro-B cell stages. The right panel diagrams the Eμ-3′RR long-distance DNA loop required for CSR.

**Figure 3:**
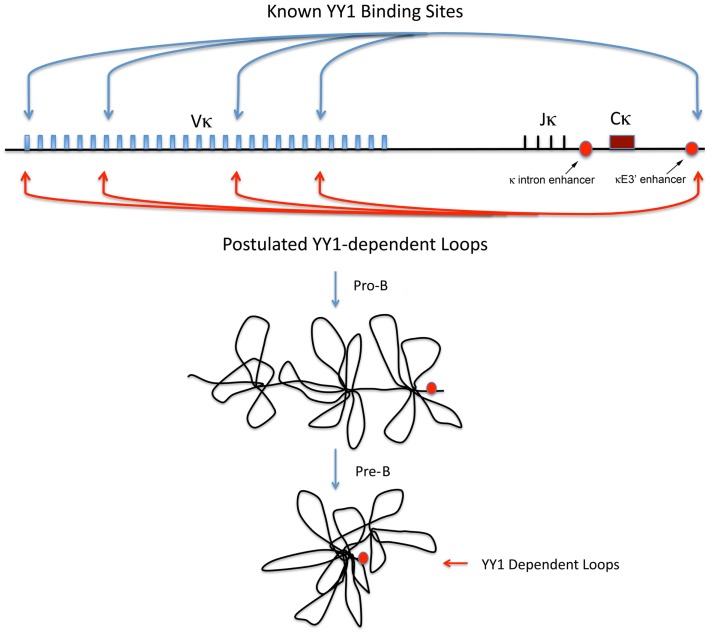
**Igκ locus diagram showing the location of known YY1 binding sites, and the postulated YY1-dependent loops required for Vκ–Jκ rearrangement**. Locations of the Igκ intron and κE3′ enhancers and shown by red circles and arrows. Postulated locus structure in pro-B and pre-B cells is show below.

Current data suggest that the Ig loci are organized as loops into rosette-like structures separated by spacer DNA (76, 78–80). A number of domains have been identified at the IgH locus, which adopt various conformations during development (76, 78–80). At the pre–pro-B cell stage, these rosette domains are in an extended conformation, but in pro-B cells the structure changes such that each V region domain is repositioned with all V_H_ regions approximately equidistant to the D_H_ and J_H_ regions, thus affording roughly equal access for recombination (79, 80) (Figure 2, left panel). Similar structures are believed to exist at the Ig kappa locus at pro-B and pre-B cell stages (Figure 3).

The mechanisms that control Ig locus contraction are unknown. A small number of transcription factors or protein complexes (YY1, Pax5, CTCF, IKAROS, cohesin, condensin, EZH2) are implicated in the DNA loops needed for V(D)J rearrangement (59, 78, 81–86), but the molecular details and regulatory processes that control this mechanism are not clear. Pax5 binds to multiple repeat sequences in the distal region of the IgH locus (PAIR sequences) and is believed to participate in rearrangement of distal V_H_ genes (83). Non-coding antisense transcripts expressed across the PAIR sequences correlate with VDJ rearrangement and are postulated to be involved with IgH locus contraction (83, 87, 88). Pax5 controls some of these transcripts (83), and recently YY1 was shown to regulate antisense transcripts across at least two PAIR sequences (87). Many Pax5 and YY1 potential binding sites exist in the IgH locus (89) and these transcription factors co-localize at some of these sites (87). Similar to the Pax5 and YY1 knock-out phenotypes (discussed below), PcG protein EZH2 knock-out results in arrest at the pro-B cell stage with impaired distal V_H_ to D_H_–J_H_ rearrangement (59). CTCF and cohesin have been argued to regulate Ig locus structure and to control interactions of D_H_ and J_H_ regions with proximal V_H_ segments and Jκ regions with proximal Vκ segments (81, 82, 90–92). Ikaros knock-out also impacts IgH rearrangement as well as locus contraction (93).

## The Role of YY1 and the REPO Domain in B Cell Development

Yin Yang 1 has long been believed to play some role in immunoglobulin (Ig) gene regulation and B cell biology because it associates with multiple Ig enhancer elements including the heavy chain intron and 3′ enhancers, the Ig kappa 3′ enhancer, as well as to a site between the C_H_ γ1 and γ2b exons (1–5, 87, 94) (Figures 2 and 3). The Shi laboratory at Harvard provided insight into the role of YY1 in B cell development by demonstrating that conditional knock-out of YY1 in the B cell lineage (using mb1-CRE which is expressed early after B lineage commitment) resulted in arrest at the pro-B cell stage (84). Pro-B cells lacking YY1 have normal D_H_–J_H_ recombination but reduced frequency of V_H_–D_H_–J_H_ recombination, with the defect being most severe for more distal V_H_ genes (84). These knock-out pro-B cells showed a defect in Ig locus contraction (84), and this phenotype has been confirmed by a number of studies (81, 88). Thus, conditional knock-out of YY1 using mb1-CRE results in arrest at the pro-B cell stage, lost Ig locus contraction, and reduced rearrangement of distal V genes. Importantly, despite the fact that proximal VDJ recombination does occur, very few mature B cells are generated in conditional knock-outs. Furthermore, introduction of a rearranged heavy chain gene only partially complements the YY1 conditional knock-out phenotype, suggesting additional roles for YY1 in early B cell development (84).

Intrigued by the similarity between the YY1 and PcG protein EZH2 B cell knock-out phenotypes (59, 84), we set out to determine the importance of YY1 PcG function for B cell development. Using YY1 wild-type and YY1ΔREPO retroviral constructs, we transduced bone marrow from *yy1^f/f^ mb1-CRE* mice and injected this transduced bone marrow into irradiated secondary recipients. Thus, within the B cell lineage of the transplanted mice, only the transduced YY1 constructs will provide YY1 function due to deletion of the endogenous *yy1* gene by mb1-CRE action. While wild-type YY1 largely restored B cell development, the YY1ΔREPO reconstituted cells arrested B cell development at the pro-B and pre-B cell stages (85). Interestingly, IgH VDJ rearrangement was largely normal, but Igκ rearrangement showed a dramatically skewed repertoire. Only a small number of Vκ genes underwent rearrangement with one third of rearrangements to the most distal 5′ V kappa gene. This dramatic result suggested that in the absence of YY1 PcG function, most of the DNA loops at the Igκ locus needed for Igκ rearrangement were abrogated, and a small number of loops that are independent of YY1 PcG function remained for Igκ Vκ–Jκ rearrangements. At least some of these loops may require E2A or Pax5 (85), although this is speculative.

## Mechanisms of Ig Locus Contraction

**Figure 4:**
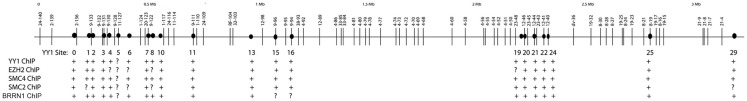
**Summary of protein co-localization data across the Igκ locus**. The Igκ locus is shown in the top panel with Vκ genes represented by vertical lines. The identified YY1 binding sites are represented by black circles. Summary of ChIP data for YY1, EZH2, SMC4, SMC2, and BRRN1 are shown in the bottom panel. Positive ChIP signals are represented by a + symbol and question marks show inconclusive ChIP data.

The dramatically skewed Vκ–Jκ rearrangement profiles in YY1ΔREPO compared to wild-type YY1 mice (85), suggested a possible direct effect of YY1 on Igκ locus structure, and loss of IgH locus contraction in a YY1 knock-out background suggested parallel effects at the heavy chain locus. Consistent with a direct effect on Igκ locus structure, RNAi knock-down of YY1 in bone marrow cultures reduced Igκ rearrangement at a subset of Vκ genes (85). Since the Shi lab showed YY1 is important for Ig locus contraction (81, 84, 88), we hypothesized that clusters of YY1 binding sites exist across the Ig loci, and that YY1 binding to these sites would result in recruitment of proteins needed for Ig locus contraction. As predicted, we identified clusters of YY1 binding sites across the Igκ locus that binds to YY1 (85). We found that PcG protein EZH2 co-localized with YY1 at these sites apparently as a result of recruitment by YY1 (85). We also identified several proteins that physically interact with the YY1 REPO domain providing potential insight into the mechanism of YY1 function in locus contraction. Intriguingly, we found that proteins from the condensin and cohesin complexes (SMC4 and SMC1) that are needed for contraction of chromosomes during mitosis (95–99), as well as lamin proteins, bind to the YY1 REPO domain. Lamin proteins are known to be involved in long-distance DNA interactions (100–103). Similarly, cohesin and condensin complexes, along with topoisomerase 2, are involved in mitotic chromosome contraction and higher order chromosome organization and dynamics (96, 104). During mitosis, condensin and cohesin proteins associate with the chromosomes and function in chromosomal contraction, cohesion, assembly, and segregation (96–98). A subpopulation of these proteins remains chromosome-associated at specific foci in the interphase nucleus (98). Importantly, cohesin and condensin proteins are involved in numerous long-distance DNA interactions (92, 105–114). Therefore, we hypothesized condensin and cohesin proteins associate with Ig loci in pro-B and pre-B cells by virtue of interaction with YY1, and thereby function to participate in Ig locus contraction. Consistent with this idea, we found that condensin proteins associate with the clusters of YY1 binding sites that we identified within the Igκ locus (85) in primary pro-B cells, but not in fibroblasts suggesting a B cell specific function of condensin and cohesin association with these sites (see Figure 4).

To test the functional consequences of YY1 and condensin binding at the Ig kappa locus, we performed RNAi knock-down and Ig kappa rearrangement assays. We found that knock-down of YY1 or condensin proteins resulted in reduced Igκ rearrangement at a subset of Vκ genes (85). Thus, YY1 binds to sites in the Ig loci, perhaps recruits PcG, condensin, cohesin, and lamin proteins to these sites, and results in specific Ig locus chromosomal contraction. The identification of condensin mutants that specifically affect T cell development supports the idea of condensin proteins (which are ubiquitously expressed) having lymphoid specific functions (115). These complexes can mediate long-distance chromosomal interactions (105, 107), and kleisin-β, a member of the condensin II complex is important for T cell development as is cohesin subunit Rad21 (92, 115). Cohesin subunit Rad 21 (a kleisin family protein) is recruited to CTCF binding sites throughout the Ig loci during B lymphocyte development (82). As condensin I is involved in the process of physically compacting DNA in the presence of hydrolyzable ATP (116), condensin complex proteins may also participate in bringing V genes in the Ig locus into close proximity with D and J gene segments.

## Long-Distance DNA Interactions and CSR

Long-distance DNA loops are also required for class switch recombination (CSR), which recombines the rearranged VDJ segments that provide antibody specificity with various Ig heavy chain constant (C) regions with different effector functions (117, 118). CSR requires a large 220 kb long-distance DNA loop synapse between the IgH intron enhancer (Eμ) region, and the 3′RR enhancer downstream of the 3′-most Cα exon (119, 120) (the Eμ-3′RR synapse; see Figure 2, right panel). In addition, CSR to individual IgH C exons requires formation of inducible DNA loops from each switch region DNA sequence into the Eμ-3′RR synapse (119, 120). Over 40 proteins are involved in the enzymology and mechanism of CSR and include DNA repair (base excision repair and mismatch repair) proteins, DNA damage sensors, factors that alter chromatin structure, factors that bind to AID, and transcriptional regulatory proteins [reviewed in Ref. (121)]. However, none of these factors are known to specifically impact the Eμ-3′RR DNA loop required for CSR.

Recent progress, however, has shed light on these long-distance DNA loops. CTCF and cohesin bind to the IgH 3′RR enhancer within the hs5–7 sites (81, 122, 123), and cohesin binding is induced at certain C_H_ switch regions in response to inducers of CSR implying a function for cohesin in CSR (123). Consistent with this, knock-down of cohesin subunits impairs CSR (123). In addition, knock-down of the cohesin loading protein NIPBL reduces CSR, reduces non-homologous end joining, and increases microhomology end joining (124). Interestingly, AID was shown to physically interact with condensin, cohesin, and INO80 complex proteins (123), precisely the same complexes that bind to YY1 (85, 125, 126).

Notably, we found that YY1 conditional knock-out in splenic B cells significantly reduces CSR (127). YY1 physically interacts with AID, leading to stabilization and increased AID nuclear accumulation, and this control of AID nuclear levels can regulate CSR. Control of nuclear levels of AID is crucial not only for regulating antibody maturation processes (CSR and somatic hypermutation), but also is important for maintaining integrity of the mammalian genome. Elevated levels of YY1 could cause aberrant accumulation of AID in germinal center B cells leading to increased mutagenesis and lymphomagenesis. Indeed, YY1 levels are elevated in germinal center-derived human DLBCL (34), suggesting that YY1 contributes to disease progression. However, we also found that YY1 has a second function important for CSR. In collaboration with Ranjan Sen (NIA), we found that YY1 is necessary for long-distance DNA loops formed between the Eμ and 3′RR enhancers (unpublished data). Recently, Kenter and colleagues identified a long-distance DNA loop between the Eμ and hs3b–hs4 sites of the 3′RR that is dramatically induced upon induction of CSR in splenic B cells (119). We found that this long-distance DNA loop is YY1-dependent (unpublished data). Thus, YY1 controls long-distance DNA loops in splenic B cells that are critical for CSR. Can the same be said of the long-distance DNA loops needed for IgH V(D)J rearrangement, and perhaps for other long-distance DNA loops? Recent evidence suggests this is the case.

## YY1-Dependent IgH Long-Distance DNA Interactions

The Sen Laboratory and colleagues indentified long-distance DNA loops in both the V_H_ distal and proximal regions, and at the 3′ end of the locus (78). They found YY1 bound to many of these segments and postulated either homotypic YY1 interactions to mediate these loops, or heterotypic interactions with other proteins (78). The essential nature of YY1 for these loops was subsequently demonstrated. In pro-B cells, YY1 conditional knock-out ablates long-distance DNA loops between the Eμ region and the distal and proximal V_H_ regions (87). In addition, YY1 knock-out in pro-B cells ablates loops between the Eμ region and the 3′RR enhancer, hs5–7 region (87). Thus, YY1 is essential for long-distance DNA loops within the IgH locus involved in either VDJ rearrangement, or CSR (Figure 2). Finally, YY1 is also involved in long-distance DNA interactions at the Th2 cytokine locus and controls IL4, IL5, and IL13 expression (128). These dramatic results indicate that YY1 is required for long-distance DNA loops that control IgH V(D)J rearrangement, CSR, and gene regulation. Our studies at the Igκ locus (85) also indicate a role for YY1 in long-distance DNA interactions needed for Igκ rearrangement (Figure 3).

## Regulatory Mechanisms for YY1 Function

How might YY1 be functioning in these diverse long-distance DNA interactions? As described above, in pro-B cells, YY1 binds constitutively to the Eμ enhancer, to hs5–7 sites in the 3′RR enhancer, to a site between the Cγ1 and Cγ2b exons, and inducibly to the hs3b site in the 3′RR enhancer in splenic B cells (5, 78, 87, 94). The mechanism of regulation of developmental stage-specific function of YY1 in VDJ rearrangement at the IgH locus (pro-B cells), in Vκ–Jκ rearrangement at the Igκ locus (pre-B cells), and in CSR at the IgH locus (mature splenic B cells), is presently unknown. YY1 may participate in regulatory stage-specific functions to control locus accessibility (129), but other factors may control accessibility enabling subsequent YY1 DNA binding.

Yin Yang 1 function can be regulated by a number of mechanisms. Stage-specific regulation could be at the level of YY1 DNA binding, such as the LPS inducible binding in the 3′RR enhancer in splenic B cells. YY1 binding to the Ig heavy chain 3′RR hypersensitive site 3b (hs3b) as well as to the Eμ enhancer is inducible by LPS (94). In this case, YY1 appears to be sequestered from DNA in resting B lymphocytes through interaction with hypophosphorylated retinoblastoma protein (Rb). However, after LPS induction, Rb becomes hyperphosphorylated and releases YY1 enabling it to bind to the hs3b and Eμ enhancers. Interestingly, hs3b and 4 hypersensitive sites are crucial for formation of Eμ: 3′RRl enhancer synapses with germline switch region promoters after cytokine treatment (119, 120). We hypothesize that LPS induction of CSR might partially result from induction of YY1 binding to the 3′RR and Eμ enhancers leading to induced DNA loop formation.

Alternatively, YY1 may be controlled by stage-specific post-translational modifications, or by stage-specific interaction with other proteins. A number of YY1 post-translational modifications can regulate YY1 DNA binding (phosphorylation of serines 180 and 184, and threonines 348 and 378) (130–132), and YY1 is sumoylated on lysine 288 (133), which can control protein–protein interactions. Phosphorylation of serines 180 and 184 is mediated by Aurora B kinase and expression of this kinase peaks in splenic germinal center B cells (www.immgen.org) when CSR is active. Several studies demonstrated that YY1 subcellular localization is regulated during cell cycle progression and development (132, 134–137) suggesting that YY1 might also regulate subcellular localization of interacting partner proteins. In addition, apoptotic stimuli promote rapid translocation of YY1 from the cytoplasm to the nucleus in asynchronous HeLa cells (138). Thus, YY1 might function to increase transport of proteins from the cytoplasm to the nucleus via the nuclear pore.

During B cell development, YY1 expression levels remain relatively constant, as defined by transcript levels (www.immgen.org). However, YY1 protein levels are regulated in some systems yielding biological responses. This is most well studied in skeletal muscle differentiation systems where YY1 expression levels drop as a result of proteolysis (24), and in cardiac disease conditions (139, 140). Thus, regulation of YY1 protein stability may control DNA loop formation.

It should be noted that RNA expression profiles of PcG proteins EZH2 and YAF2, as well as cohesin, and condensin subunit proteins SMC4, SMC2, SMC1, SMC3, CAP-G, CAP-H (BRRN1), and CAP-D2 all peak during B cell development at the pre-B cell stage (www.immgen.org). Expression levels are also high in pro-B cells, but peak in pre-B cells, then drop in immature B cell stages. This expression pattern is coincident with the timing of Ig rearrangement and is consistent with a role in Ig locus contraction and rearrangement. However, this timing is also coincident with high levels of proliferation in pre-B cells suggesting a possible effect of YY1 on the pre-B proliferative burst during development. All factors peak again in germinal center B cells (www.immgen.org) suggesting possible roles in proliferation, CSR, or somatic hypermutation.

Whatever the mode of locus accessibility or YY1 DNA binding, YY1 may then recruit proteins to DNA that are required for long-distance DNA interactions. As presented above, YY1 physically interacts with PcG, condensin, cohesin, and lamin proteins, all involved in long-distance DNA interactions, and we have noted co-localization of some of these proteins with YY1 at the Igκ locus (Figure 4). PcG proteins can mediate long-distance DNA interactions (60), and since YY1 recruits PcG proteins to DNA via the REPO domain (50, 61), we predict that this interaction will be important for long-distance interactions leading to DNA loop formation. Notably, condensin and cohesin complex proteins (105, 107), and lamin proteins (100–103) are all involved in long-distance DNA structures, suggesting that the DNA binding capacity of YY1 at IgH and Igκ sequences may nucleate protein–protein interactions that govern DNA looping mechanisms. In addition to co-localization of YY1 and condensin proteins at the Igκ locus, YY1 co-localizes with cohesin at the hs5–7 sites in the 3′RR enhancer (78, 81).

## Models of YY1-Mediated Long-Distance DNA Interactions

**Figure 5:**
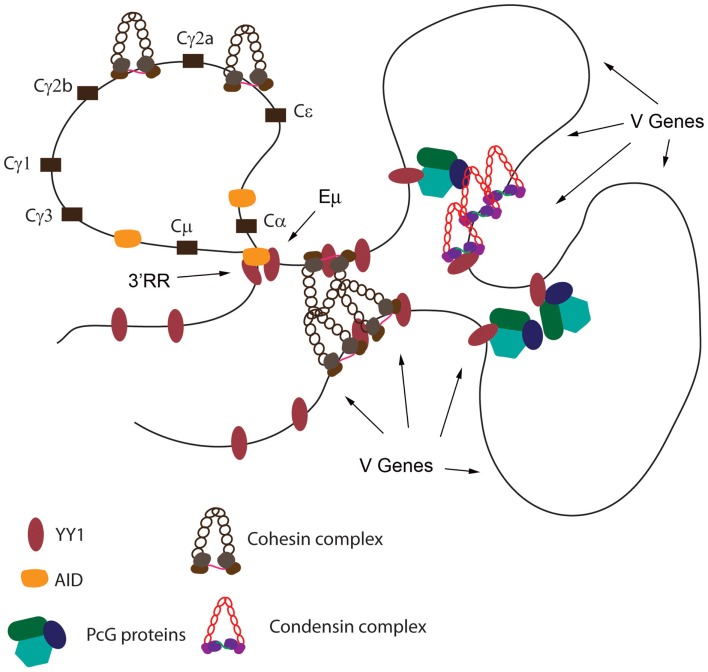
**Model of YY1 recruitment of proteins to DNA needed for long-distance DNA interactions**. Using the IgH locus as a model, YY1 binding sites are indicated. Binding by YY1 then results in recruitment of condensin, cohesin, and PcG complex proteins. These proteins may form homotypic or heterotypic interactions to mediate long-distance DNA interactions. Positions of the Eμ enhancer, 3′RR enhancer, and various V genes are shown by arrows. Black rectangles represent various C_H_ constant regions. YY1 also physically interacts with AID, and AID is able to interact with condensin, and cohesin complexes, and thus may contribute to DNA loop formation in germinal center B cells.

Based upon: (a) the crucial nature of the YY1 REPO domain for B cell development, (b) the ability of this domain to recruit PcG proteins to DNA, (c) the physical interaction of the REPO domain with PcG, condensin, cohesin, and lamin proteins, (d) the co-localization of YY1, EZH2, and condensin proteins across the Igκ locus, (e) the co-localization of YY1 and cohesin proteins at the IgH 3′RR enhancer, (f) the effect of cohesin knock-down on CSR, (g) the effect of condensin subunit knock-down on Vκ–Jκ rearrangement, (h) the high levels of EZH2, YAF2, cohesin, and condensin proteins in pro-B, pre-B, and germinal center cells, (i) the critical role of YY1 in long-distance DNA loops in the IgH V region and 3′ region, and (j) the regulatory role of YY1 in CSR, we propose the following mechanism. We propose that YY1 binds to sites spanning the IgH and Igκ loci. Concomitant with YY1 DNA binding, increased EZH2, YAF2, cohesin, and condensin subunit expression results in these proteins binding to the same DNA regions, presumably due to interactions with YY1. The nucleated PcG, cohesin, and condensin proteins then mediate long-distance interactions between the YY1 binding sites resulting in contraction of the Ig loci in looped or rosette structures (Figure 5). These loops then control somatic rearrangement of IgH and Igκ genes as well as CSR. Immediately upon maturation to the immature B cell stage, or upon maturation to plasma cells, EZH2, YAF2, cohesin, and condensin protein expression drops dramatically (www.immgen.org), thus facilitating de-contraction of the Ig loci, perhaps assisting in regulation of the allelic exclusion process, and causing a decrease in the inducible loops needed for CSR. In the case of CSR, it is intriguing that AID binds to many of the same factors that bind to YY1 (condensin, cohesin, and INO80 complexes) (123). Thus, YY1–AID physical interaction may also contribute to DNA loop formation (Figure 5).

Finally, it has been proposed that YY1 function in long-distance DNA interactions relates to the regulation of non-coding antisense transcripts in the IgH V_H_ PAIR sequences (88). YY1 knock-out ablates some of these transcripts, and these transcripts have been proposed to play a role in IgH locus contraction (87, 88). Some RNA transcripts are known to regulate long-distance DNA interactions via interactions with the mediator complex (141). Whether YY1 functions in this mechanism is presently unclear.

## Future Studies and Remaining Questions

A number of outstanding questions remain. (1) Is recruitment to DNA of proteins involved in DNA loop formation dependent upon YY1 DNA binding? (2) What mechanisms enable YY1 to function at distinct loci at various developmental stages? (3) Is YY1 function controlled by post-translational modifications? (4) Is YY1 controlled by stage-specific protein interactions? (5) What functions and domains of YY1 are needed for DNA looping, V(D)J rearrangement, and CSR? (6) What are the biochemical mechanisms for Ig locus contraction and for DNA loop formation? These questions and others are important for immune function and control of gene expression. The ubiquitous nature of YY1 and its involvement in looping at multiple loci (78, 87, 128) suggests that paradigms learned in the Ig systems will be globally applicable to other long-distance DNA interactions.

## Conflict of Interest Statement

The author declares that the research was conducted in the absence of any commercial or financial relationships that could be construed as a potential conflict of interest.

## References

[1] Church GM Ephrussi A Gilbert W Tonegawa S Cell-type-specific contacts to immunoglobulin enhancers in nuclei. Nature (1985) 313:798–801 10.1038/313798a0 3919308

[2] Ephrussi A Church GM Tonegawa S Gilbert W B lineage-specific interactions of an immunoglobulin enhancer with cellular factors in vivo. Science (1985) 227:134–40 10.1126/science.3917574 3917574

[3] Weinberger J Baltimore D Sharp PA Distinct factors bind to apparently homologous sequences in the immunoglobulin heavy-chain enhancer. Nature (1986) 322:846–8 10.1038/322846a0 3092107

[4] Sen R Baltimore D Multiple nuclear factors interact with the immunoglobulin enhancer sequences. Cell (1986) 46:705–16 10.1016/0092-8674(86)90346-6 3091258

[5] Park K Atchison ML Isolation of a candidate repressor/activator, NF-E1 (YY-1, δ), that binds to the immunoglobulin κ 3’ enhancer and the immunoglobulin heavy-chain μE1 site. Proc Natl Acad Sci U S A (1991) 88:9804–8 10.1073/pnas.88.21.9804 1946405 PMC52809

[6] Shi Y Seto E Chang L-S Shenk T Transcriptional repression by YY1, a human GLI-Kruppel-related protein, and relief of repression by adenovirus E1A protein. Cell (1991) 67:377–88 10.1016/0092-8674(91)90189-6 1655281

[7] Hariharan N Kelley DE Perry RP δ, a transcription factor that binds to downstream elements in several polymerase II promoters, is a functionally diverse zinc finger protein. Proc Natl Acad Sci U S A (1991) 88:9799–803 10.1073/pnas.88.21.9799 1946404 PMC52808

[8] Flanagan JR Becker KG Ennist DL Gleason SL Driggers PH Levi B-Z Cloning of a negative transcription factor that binds to the upstream conserved region of Moloney murine leukemia virus. Mol Cell Biol (1992) 12:38–44 1309593 10.1128/mcb.12.1.38-44.1992 PMC364067

[9] Bushmeyer S Park K Atchison ML Characterization of functional domains within the multifunctional transcription factor, YY1. J Biol Chem (1995) 270:30213–20 10.1074/jbc.270.50.30213 8530432

[10] Bushmeyer SM Atchison ML Identification of YY1 sequences necessary for association with the nuclear matrix and for transcriptional repression functions. J Cell Biochem (1998) 68:484–99 10.1002/(SICI)1097-4644(19980315)68:4<484::AID-JCB8>3.3.CO;2-F 9493911

[11] Galvin KM Shi Y Multiple mechanisms of transcriptional repression by YY1. Mol Cell Biol (1997) 17:3723–32 9199306 10.1128/mcb.17.7.3723 PMC232224

[12] Lee J-S Galvin KM See RH Eckner R Livingston D Moran E Relief of YY1 transcriptional repression by adenovirus E1A is mediated by EIA-associated protein p300. Genes Dev (1995) 9:1188–98 10.1101/gad.9.10.1188 7758944

[13] Lee T-C Zhang Y Schwartz RJ Bifunctional transcriptional properties of YY1 in regulating muscle actin and c-myc gene expression during myogenesis. Oncogene (1994) 9:1047–52 8134108

[14] Lewis BA Tullis G Seto E Horikoshi N Weinmann R Shenk T Adenovirus E1A protein interact with the cellular YY1 transcription factor. J Virol (1995) 69:1628–36 7853498 10.1128/jvi.69.3.1628-1636.1995 PMC188760

[15] Yang W-M Inouye C Zeng Y Bearss D Seto E Transcriptional repression by YY1 is mediated by interaction with a mammalian homolog of the yeast global regulator RPD3. Proc Natl Acad Sci U S A (1996) 93:12845–50 10.1073/pnas.93.23.12845 8917507 PMC24008

[16] Thomas MJ Seto E Unlocking the mechanisms of transcription factor YY1: are chromatin modifying enzymes the key? Gene (1999) 236:197–208 10.1016/S0378-1119(99)00261-9 10452940

[17] Austen M Luscher B Luscher-Firzlaff JM Characterization of the transcriptional regulator YY1. The bipartite transactivation domain is independent of interaction with the TATA box-binding protein, transcription factor IIB, TAFII55, or cAMP-responsive element-binding protein (CPB)-binding protein. J Biol Chem (1997) 272:1709–17 8999850 10.1074/jbc.272.3.1709

[18] Donohoe ME Zhang X McGinnis L Biggers J Li E Shi Y Targeted disruption of mouse Yin Yang 1 transcription factor results in peri-implantation lethality. Mol Cell Biol (1999) 19:7237–44 10490658 10.1128/mcb.19.10.7237 PMC84716

[19] Gregoire S Karra R Passer D Deutsch MA Krane M Feistritzer R Essential and unexpected role of Yin Yang 1 to promote mesodermal cardiac differentiation. Circ Res (2013) 112:900–10 10.1161/CIRCRESAHA.113.259259 23307821 PMC3629954

[20] Lu L Sun K Chen X Zhao Y Wang L Zhou L Genome-wide survey by ChIP-seq reveals YY1 regulation of lincRNAs in skeletal myogenesis. EMBO J (2013) 32:2575–88 10.1038/emboj.2013.182 23942234 PMC3791367

[21] Petkova V Romanowski MJ Sulijoadikusumo I Rohne D Kang P Shenk T Interaction between YY1 and the retinoblastoma protein. J Biol Chem (2001) 276:7932–6 10.1074/jbc.M007411200 11118439

[22] Schlisio S Halperin T Vidal M Nevins JR Interaction of YY1 with E2Fs, mediated by RYBP, provides a mechanism for specificity of E2F function. EMBO J (2002) 21:5775–86 10.1093/emboj/cdf577 12411495 PMC131074

[23] Sucharov CC Dockstader K McKinsey TA YY1 protects cardiac myocytes from pathologic hypertrophy by interacting with HDAC5. Mol Biol Cell (2008) 19:4141–53 10.1091/mbc.E07-12-1217 18632988 PMC2555926

[24] Walowitz JL Bradley ME Chen S Lee T Proteolytic regulation of the zinc finger transcription factor YY1, a repressor of muscle-restricted gene expression. J Biol Chem (1998) 273:6656–61 10.1074/jbc.273.12.6656 9506962

[25] Erkeland S Valkhof M Heijmans-Antonissen C Delwel R Valk PJ Hermans MH The gene encoding transcriptional regulator Yin Yang 1 (YY1) is a myeloid transforming gene interfering with neutrophilic differentiation. Blood (2003) 101:1111–7 10.1182/blood-2002-04-1207 12393438

[26] Gabellini D Greeen MR Tupier R Inappropriate gene activation in FSHD: a repressor complex binds a chromosomal repeat deleted in dystrophic muscle. Cell (2002) 110:339–48 10.1016/S0092-8674(02)00826-7 12176321

[27] Li J Shen H Himmel KL Dupuy AJ Largaespada DA Nakamura T Leukaemia disease genes: large scale cloning and pathway predictions. Nat Genet (1999) 23:348–53 10.1038/15531 10610183

[28] Green MR Monti S Dalla-Favera R Pasqualucci L Walsh NC Schmidt-Supprian M Signatures of murine B-cell development implicate Yy1 as a regulator of the germinal center-specific program. Proc Natl Acad Sci U S A (2011) 108:2873–8 10.1073/pnas.1019537108 21282644 PMC3041080

[29] Duke JL Liu M Yaari G Khalil AM Tomayko MM Shlomchik MJ Multiple transcription factor binding sites predict AID targeting in non-Ig genes. J Immunol (2013) 190:3878–88 10.4049/jimmunol.1202547 23514741 PMC3689293

[30] Gordon S Akopyan G Garban H Bonavida B Transcription factor YY1: structure, function, and therapeutic implications in cancer biology. Oncogene (2006) 25:1125–42 10.1038/sj.onc.1209080 16314846

[31] Sui G The regulation of YY1 in tumorigenesis and its targeting potential in cancer therapy. Mol Cell Pharmacol (2009) 1:157–76 10.4255/mcpharmacol.09.20

[32] Zaravinos A Spandidos DA Yin Yang 1 expression in human tumors. Cell Cyle (2010) 9:512–22 10.4161/cc.9.3.10588 20081375

[33] Castellano G Torrisi E Ligresti G Malaponte G Militello L Russo AE The involvement of the transcription factor Yin Yang 1 in cancer development and progression. Cell Cycle (2009) 8:1367–72 10.4161/cc.8.9.8314 19342874

[34] Castellano G Torrisi E Ligresti G Nicoletti F Malaponte G Travali S Yin Yang 1 overexpression in diffuse large B-cell lymphoma is associated with B-cell transformation and tumor progression. Cell Cycle (2010) 9:557–63 10.4161/cc.9.3.10554 20081364

[35] Donohoe ME Zhang L-F Xu N Shi Y Lee JT Identification of a Ctcf cofactor, YY1, for the X chromosome binary switch. Mol Cell (2007) 25:43–56 10.1016/j.molcel.2006.11.017 17218270

[36] Jeon Y Lee JT YY1 tethers Xist RNA to the inactive X nucleation center. Cell (2011) 146:119–33 10.1016/j.cell.2011.06.026 21729784 PMC3150513

[37] Kim JD Hinz AK Choo JH Stubbs L Kim J YY1 as a controlling factor for the Peg3 and Gnas imprinted domains. Genomics (2007) 89:262–9 10.1016/j.ygeno.2006.09.009 17067777 PMC1828871

[38] Coull JJ He G Rucker MC Dervan PB Margolis DM Targeted derepression of the human immunodeficiency virus type 1 long terminal repeat by pyrrole-imidazole polyamines. J Virol (2002) 76:12349–54 10.1128/JVI.76.23.12349-12354.2002 12414976 PMC136904

[39] Dong XP Stubenrauch F Beyer-Finkler E Pfister H Prevalence of deletions of YY1-binding sites in episomal HPV16 DNA from cervical cancers. Int J Cancer (1994) 58:803–8 10.1002/ijc.2910580609 7927871

[40] He G Margolis DM Counterregulation of chromatin deacetylation and histone deacetylase occupancy at the integrated promoter of human immunodeficiency virus type 1 (HIV-1) by the HIV-1 repressor YY1 and HIV-1 activator Tat. Mol Cell Biol (2002) 22:2965–73 10.1128/MCB.22.9.2965-2973.2002 11940654 PMC133763

[41] Margolis DM Somasundaran M Green MR Human transcription factor YY1 represses human immunodeficiency virus type 1 transcription and virion production. J Virol (1994) 68:905–10 8289393 10.1128/jvi.68.2.905-910.1994 PMC236527

[42] May M Dong XP Beyer-Finkler E Stubenrauch F Fuchs PG Pfister H The E6/E7 promoter of extrachromosomal HPV16 DNA in cervical cancers escapes from cellular repression by mutation of target sequences for YY1. EMBO J (1994) 13:1460–6 8137827 10.1002/j.1460-2075.1994.tb06400.x PMC394965

[43] Moriuchi M Moriuchi H YY1 transcription factor down-regulates expression of CCR5, a major coreceptor for HIV-1. J Biol Chem (2003) 278:13003–7 10.1074/jbc.M204980200 12571248

[44] Schmidt M Kedzia W Gozdzicka-Jozefiak A Intratype HPV16 sequence variation within LCR of isolates from asymptomatic carriers of cervical cancers. J Clin Virol (2001) 23:65–77 10.1016/S1386-6532(01)00189-5 11595585

[45] Tan SH Baker CC Stunkel W Bernard HU A transcriptional initiator overlaps with a conserved YY1 binding site in the long control region of human papillomavirus type16. Virol (2003) 305:486–501 10.1006/viro.2002.1779 12573593

[46] Veress G Murvai M Szarka K Juhasz A Konya J Gergely L Transcriptoinal activity of human papillomavirus type 16 variants having deletions in the long control region. Eur J Cancer (2001) 37:1946–52 10.1016/S0959-8049(01)00222-2 11576852

[47] Brown JL Mucci D Whiteley M Dirksen M-L Kassis JA The *Drosophila* Polycomb group gene pleiohomeotic encodes a DNA binding protein with homology to the transcription factor YY1. Mol Cell (1998) 1:1057–64 10.1016/S1097-2765(00)80106-9 9651589

[48] Girton JR Jeon SH Novel embryonic and adult homeotic phenotypes are produced by pleiohomeotic mutations in *Drosophila*. Dev Biol (1994) 161:393–407 10.1006/dbio.1994.1040 8313991

[49] Atchison L Ghias A Wilkinson F Bonini N Atchison ML The YY1 transcription factor functions as a PcG protein in vivo. EMBO J (2003) 22:1347–58 10.1093/emboj/cdg124 12628927 PMC151054

[50] Srinivasan L Atchison ML YY1 DNA binding and PcG recruitment requires CtBP. Genes Dev (2004) 18:2596–601 10.1101/gad.1228204 15520279 PMC525539

[51] Srinivasan L Pan X Atchison ML Transient requirements of YY1 expression for PcG transcriptional rescue and phenotypic rescue. J Cell Biochem (2005) 96:689–99 10.1002/jcb.20562 16052488

[52] Basu A Wilkinson FH Colavita K Fennelly C Atchison ML YY1 DNA binding and interaction with YAF2 is essential for Polycomb recruitment. Nucleic Acids Res (2013) 272(3):1709–17 10.1093/nar/gkt1187 24285299 PMC3936737

[53] Caretti G Di Padova M Micales B Lyons GE Sartorelli V The Polycomb Ezh2 methyltransferase regulates muscle gene expression and skeletal muscle differentiation. Genes Dev (2004) 18:2627–38 10.1101/gad.1241904 15520282 PMC525543

[54] Sing A Pannell D Karaiskakis A Sturgeon K Djabali M Ellis J A vertebrate Polycomb response element governs segmentation of the posterior hindbrain. Cell (2009) 138:885–97 10.1016/j.cell.2009.08.020 19737517

[55] Woo CJ Kharchenko PV Daheron L Park PJ Kingston RE Variable requirements for DNA-binding proteins at polycomb-dependent repressive regions in the human HOX clusters. Mol Cell Biol (2013) 33:3274–85 10.1128/MCB.00275-13 23775117 PMC3753906

[56] Mendenhall EM Koche RP Truong T Zhou VW Issac B Chi AS GC-rich sequence elements recruit PRC2 in mammalian ES cells. PLoS Genet (2010) 6:e1001244 10.1371/journal.pgen.1001244 21170310 PMC3000368

[57] Okulski H Druck B Bhalerao S Ringrose L Quantitative analysis of polycomb response elements (PREs) at identical genomic locations distinguishes contributions of PRE sequence and genomic environment. Epigenetics Chromatin (2011) 4:4 10.1186/1756-8935-4-4 21410956 PMC3070613

[58] Ringrose L Rehmsmeier M Dura J-M Paro R Genome-wide prediction of Polycomb/Trithorax response elements in *Drosophila melanogaster*. Dev Cell (2003) 5:759–71 10.1016/S1534-5807(03)00337-X 14602076

[59] Su I-H Basavaraj A Krutchinsky AN Hobert O Ullrich A Chait BT Ezh2 controls B cell development through histone H3 methylation and *Igh* rearrangement. Nat Immunol (2003) 4:124–31 10.1038/ni876 12496962

[60] Lanzuolo C Roure V Dekker J Bantignies F Orlando V Polycomb response elements mediate the formation of chromosome higher-order structures in the bithorax complex. Nat Cell Biol (2007) 9:1167–74 10.1038/ncb1637 17828248

[61] Wilkinson FH Park K Atchison ML Polycomb recruitment to DNA in vivo by the YY1 REPO domain. Proc Natl Acad Sci U S A (2006) 103:19296–301 10.1073/pnas.0603564103 17158804 PMC1748220

[62] Garcia E Marcos-Gutierrez C del Mar Lorente M Moreno JC Vidal M RYBP, a new repressor protein that interacts with components of the mammalian Polycomb complex, and with the transcription factor YY1. EMBO J (1999) 18:3404–18 10.1093/emboj/18.12.3404 10369680 PMC1171420

[63] Kalenik JL Chen D Bradley ME Chen SJ Lee TC Yeast two-hybrid cloning of a novel zinc finger protein that interacts with the multifunctional transcription factor YY1. Nucleic Acids Res (1997) 25:843–9 10.1093/nar/25.4.843 9016636 PMC146511

[64] Trojer P Cao AR Gao Z Li Y Zhang J Xu X L3MBTL2 protein acts in concert with PcG protein-mediated monoubiquitination of H2A to establish a repressive chromatin structure. Mol Cell (2011) 42:438–50 10.1016/j.molcel.2011.04.004 21596310 PMC3142354

[65] Woo CJ Kharchenko PV Daheron L Park PJ Kingston RE A region of the human HOXD cluster that confers polycomb-group responsiveness. Cell (2010) 140:99–110 10.1016/j.cell.2009.12.022 20085705 PMC3324942

[66] Wilkinson F Pratt H Atchison ML PcG recruitment by the YY1 REPO domain can be mediated by Yaf2. J Cell Biochem (2010) 109:478–86 10.1002/jcb.22424 19960508 PMC3677512

[67] Cobb RM Oestreich KJ Osipovich OA Oltz EM Accessibility control of V(D)J recombination. Adv Immunol (2006) 91:45–109 10.1016/S0065-2776(06)91002-5 16938538

[68] Fugmann SD Lee AI Shockett PE Villey IJ Schatz DG The RAG proteins and V(D)J recombination: complexes, ends, and transposition. Annu Rev Immunol (2000) 18:495–527 10.1146/annurev.immunol.18.1.495 10837067

[69] Jung D Giallourakis C Mostoslavsky R Alt FW Mechanism and control of V(D)J recombination at the immunoglobulin heavy chain locus. Annu Rev Immunol (2006) 24:541–70 10.1146/annurev.immunol.23.021704.115830 16551259

[70] Subrahmanyam R Sen R RAGs’ eye view of the immunoglobulin heavy chain locus. Semin Immunol (2010) 22:337–45 10.1016/j.smim.2010.08.003 20864355

[71] Fuxa M Skok J Souabni A Salvagiotto G Roldan E Busslinger M Pax5 induces V-to-DJ rearrangements and locus contraction of the immunoglobulin heavy-chain gene. Genes Dev (2004) 18:411–22 10.1101/gad.291504 15004008 PMC359395

[72] Kosak ST Skok JA Medina KL Riblet R Le Beau MM Fisher AG Subnuclear compartmentalization of immunoglobulin loci during lymphocyte development. Science (2002) 296:158–62 10.1126/science.1068768 11935030

[73] Roldán E Fuxa M Chong W Martinez D Novatchkova M Busslinger M Locus “decontraction” and centromeric recruitment contribute to allelic exclusion of the immunoglobulin heavy-chain gene. Nat Immunol (2005) 6:31–41 10.1038/ni1150 15580273 PMC1592471

[74] Skok JA Brown KE Azuara V Caparros M-L Baxter J Takacs K Non equivalent nuclear location of immunoglobulin alleles in B lymphocytes. Nat Immunol (2001) 2:848–54 10.1038/ni0901-848 11526401

[75] Hesslein DG Pflugh DL Chowdhury D Bothwell AL Sen R Schatz DG Pax5 is required for recombination of transcribed, acetylated, 5’ IgH V gene segments. Genes Dev (2003) 17:37–42 10.1101/gad.1031403 12514097 PMC195966

[76] Jhunjhunwala S van Zelm MC Peak MM Murre C Chromatin architecture and the generation of antigen receptor diversity. Cell (2009) 138:435–48 10.1016/j.cell.2009.07.016 19665968 PMC2726833

[77] Sayegh C Jhunjhunwala S Riblet R Murre C Visualization of looping involving the immunoglobulin heavy-chain locus in developing B cells. Genes Dev (2005) 19:322–7 10.1101/gad.1254305 15687256 PMC546510

[78] Guo C Gerasimova T Hao H Ivanova I Chakraborty T Selimyan R Two forms of loops generate the chromatin conformation of the immunoglobulin heavy-chain gene locus. Cell (2011) 147:332–43 10.1016/j.cell.2011.08.049 21982154 PMC3685183

[79] Jhunjhunwala S van Zelm MC Peak MM Cutchin S Riblet R van Dogen JJ The 3D structure of the immunoglobulin heavy-chain locus: implications for long-range genomic interactions. Cell (2008) 133:265–79 10.1016/j.cell.2008.03.024 18423198 PMC2771211

[80] Lucas JS Bossen C Murre C Transcription and recombination factories: common features? Curr Opin Cell Biol (2010) 23:1–7 10.1016/j.ceb.2010.11.007 21169003 PMC3070187

[81] Degner SC Verma-Gaur J Wong TP Bossen C Iverson GM Torkamani A CCCTC-binding factor (CTCF) and cohesin influence the genomic architecture of the Igh locus and antisense transcription in pro-B cells. Proc Natl Acad Sci U S A (2011) 108:9566–71 10.1073/pnas.1019391108 21606361 PMC3111298

[82] Degner SC Wong TP Jankevicius G Feeney AJ Cutting edge: developmental stage-specific recruitment of cohesin to CTCF sites throughout immunoglobulin loci during B lymphocyte development. J Immunol (2009) 182:44–8 19109133 10.4049/jimmunol.182.1.44 PMC2625297

[83] Ebert A McManus S Tagoh H Medvedovic J Slavagiotto G Novatchkova M The distal VH gene cluster of the Igh locus contains distinct regulatory elements with Pax5 transcription factor-dependent activity in pro-B cells. Immunity (2011) 34:175–87 10.1016/j.immuni.2011.02.005 21349430

[84] Liu H Schmidt-Supprain M Shi Y Hobeika E Barteneva N Jumaa H Yin Yang 1 is a critical regulator of B-cell development. Genes Dev (2007) 21:1179–89 10.1101/gad.1529307 17504937 PMC1865490

[85] Pan X Papasani M Hao Y Calamito M Wei F Quinn WJ YY1 controls Igκ repertoire and B-cell development, and localizes with condensin on the Igκ locus. EMBO J (2013) 32:1168–82 10.1038/emboj.2013.66 23531880 PMC3630362

[86] Ribeiro de Almeida C Stadhouders R de Bruijn MJ Bergen IM Thongjuea S Lenhard B The DNA-binding protein CTCF limits proximal Vκ recombination and restricts κ enhancer interactions to the immunoglobulin κ light chain locus. Immunity (2011) 35:501–13 10.1016/j.immuni.2011.07.014 22035845

[87] Medvedovic J Ebert A Tagoh H Tamir IM Schwickert TA Novatchkova M Flexible long-range loops in the VH gene region of the Igh locus facilitate the generation of a diverse antibody repertoire. Immunity (2013) 39:229–44 10.1016/j.immuni.2013.08.011 23973221 PMC4810778

[88] Verma-Gaur J Torkamani A Schaffer L Head SR Schork NJ Feeney AJ Noncoding transcription within the Igh distal V(H) region at PAIR elements affects the 3D structure of the Igh locus in pro-B cells. Proc Natl Acad Sci U S A (2012) 109:17004–9 10.1073/pnas.1208398109 23027941 PMC3479473

[89] Calame K Atchison M YY1 helps bring loose ends together. Genes Dev (2007) 21:1145–52 10.1101/gad.1559007 17504933

[90] Feeney AJ Verma-Gaur J CTCF-cohesin complex: architect of chromatin structure regulates V(D)J rearrangement. Cell Res (2012) 22:280–2 10.1038/cr.2011.188 22105486 PMC3271585

[91] Guo C Yoon HS Franklin A Jain S Ebert A Cheng H-L CTCF-binding elements mediate control of V(D)J recombination. Nature (2011) 477:424–30 10.1038/nature10495 21909113 PMC3342812

[92] Seitan VC Hao B Tachibana-Konwalski K Lavagnolli T Mira-Bontenbal H Brown KW A role for cohesin in T-cell-receptor rearrangement and thymocyte differentiation. Nature (2011) 476:467–71 10.1038/nature10312 21832993 PMC3179485

[93] Reynaud D Demarco IA Reddy KL Schjerven H Bertolino E Chen Z Regulation of B cell fate commitment and immunoglobulin heavy-chain gene rearrangements by Ikaros. Nat Immunol (2008) 9:927–36 10.1038/ni.1626 18568028 PMC2699484

[94] Gordon SJ Saleque S Birshtein BK Yin Yang 1 is a lipopolysaccharide-inducible activator of the murine 3’ Igh enhancer, hs3. J Immunol (2003) 170:5549–57 12759432 10.4049/jimmunol.170.11.5549

[95] Kimura K Cuvier O Hirano T Chromosome condensation by a human condensin complex in *Xenopus* egg extracts. J Biol Chem (2001) 276:5417–20 10.1074/jbc.C000873200 11136719

[96] Lehmann AR The role of SMC proteins in the responses to DNA damage. DNA Repair (2005) 4:309–14 10.1016/j.dnarep.2004.07.009 15661654

[97] Savvidou E Cobbe N Steffensen S Cotterill S Heck MM *Drosophila* CAP-D2 is required for condensin complex stability and resolution of sister chromatids. J Cell Sci (2005) 118:2529–43 10.1242/jcs.02392 15923665

[98] Schmiesing JA Gregson HC Zhou S Yokomori K A human condensin complex containing hCAP-C-hCAP-E and CNAP1, a homolog of *Xenopus* XCAP-D2, colocalizes with phosphorylated histone H3 during the early stage of mitotic chromosome condensation. Mol Cell Biol (2000) 20:6996–7006 10.1128/MCB.20.18.6996-7006.2000 10958694 PMC88774

[99] Steffensen S Coelho PA Cobbe N Vass S Costa M Hassan B A role for *Drosophila* SMC4 in the resolution of sister chromatids in mitosis. Curr Biol (2001) 11:295–307 10.1016/S0960-9822(01)00096-3 11267866

[100] Guelen L Pagie L Brasset E Meuleman W Faza MB Talhout W Domain organization of human chromosomes revealed by mapping of nuclear lamina interactions. Nature (2008) 453:948–51 10.1038/nature06947 18463634

[101] Peric-Hupkes D Meuleman W Pagie L Bruggeman SW Solovei I Brugman W Molecular maps of the reorganization of genome-nuclear lamina interactions during differentiation. Mol Cell (2010) 38:603–13 10.1016/j.molcel.2010.03.016 20513434 PMC5975946

[102] Pickersgill H Kalverda B de Wit E Talhout W Fornerod M van Steensel B Characterization of the *Drosophila melanogaster* genome at the nuclear lamina. Nat Genet (2006) 38:1005–14 10.1038/ng1852 16878134

[103] van Bemmel JG Pagie L Braunschweig U Brugman W Meuleman W Kerkhoven RM The insulator protein SU(HW) fine-tunes nuclear lamina interactions of the *Drosophila* genome. PLoS One (2010) 5:e15013 10.1371/journal.pone.0015013 21124834 PMC2991331

[104] Losada A Hirano T Dynamic molecular linkers of the genome: the first decade of SMC proteins. Genes Dev (2005) 19:1269–87 10.1101/gad.1320505 15937217

[105] D’Ambrosio D Schmidt CK Katou Y Kelly G Itoh T Shirahige K Identification of cis-acting sites of condensin loading onto budding yeast chromosomes. Genes Dev (2008) 22:2215–27 10.1101/gad.1675708 18708580 PMC2518811

[106] Haering CH Forward: the many fascinating functions of SMC protein complexes. Chromosome Res (2009) 17:127–9 10.1007/s10577-008-9008-8 19308695

[107] Haeusler RA Pratt-Hyatt M Good PD Gipson TA Engelke DR Clustering of yeast tRNA genes is mediated by specific association of condensin with tRNA gene transcription complexes. Genes Dev (2008) 22:2204–14 10.1101/gad.1675908 18708579 PMC2518813

[108] Hudson DF Marshall KM Earnshaw WC Condensin: architect of mitotic chromosomes. Chromosome Res (2009) 17:131–44 10.1007/s10577-008-9009-7 19308696

[109] Hudson DF Ohta S Freisinger T MacIsaac F Sennels L Alves F Molecular and genetic analysis of condensin function in vertebrate cells. Mol Biol Cell (2008) 19:3070–9 10.1091/mbc.E08-01-0057 18480406 PMC2441691

[110] Hudson DF Vagnarelli P Gassmann R Earnshaw WC Condensin is required for nonhistone protein assembly and structural integrity of vertebrate mitotic chromosomes. Dev Cell (2003) 5:323–36 10.1016/S1534-5807(03)00199-0 12919682

[111] Kagey MH Newman JJ Bilodeau S Zhan Y Orlando DA van Berkum NL Mediator and cohesin connect gene expression and chromatin architecture. Nature (2010) 467:430–5 10.1038/nature09380 20720539 PMC2953795

[112] Phillips-Cremins JE Sauria ME Sanyal A Gerasimova TI Lajoie BR Bell JS Architectural protein subclasses shape 3D organization of genomes during lineage commitment. Cell (2013) 153:1281–95 10.1016/j.cell.2013.04.053 23706625 PMC3712340

[113] Wei Z Gao F Kim S Yang H Lyu J An W Klf4 organizes long-range chromosomal interactions with the Oct4 locus in reprogramming and pluripotency. Cell Stem Cell (2013) 12:36–47 10.1016/j.stem.2013.05.010 23747203

[114] Zhang H Jiao W Sun L Fan J Chen M Wang H Intrachromosomal looping is required for activation of endogenous pluripotency genes during reprogramming. Cell Stem Cell (2013) 13:30–5 10.1016/j.stem.2013.05.012 23747202

[115] Gosling KM Makaroff LE Theodoratos A Kim Y-H Whittle B Rui L A mutation in a chromosome condensin II subunit, kleisin β, specifically disrupts T cell development. Proc Natl Acad Sci U S A (2007) 104:12445–50 10.1073/pnas.0704870104 17640884 PMC1941488

[116] Strick TR Kawaguchi T Hirano T Real-time detection of single-molecule DNA compaction by condensin I. Curr Biol (2004) 14:874–80 10.1016/j.cub.2004.04.038 15186743

[117] Peled JU Kuang FL Iglesias-Ussel MD Roa S Kalis SL Goodman MF The biochemistry of somatic hypermutation. Annu Rev Immunol (2008) 26:481–511 10.1146/annurev.immunol.26.021607.090236 18304001

[118] Stavnezer J Guikema JE Schrader CE Mechanism and regulation of class switch recombination. Annu Rev Immunol (2008) 26:261–92 10.1146/annurev.immunol.26.021607.090248 18370922 PMC2707252

[119] Kumar S Wuerffel R Achour I Lajoie B Sen R Dekker J Flexible ordering of antibody class switch and V(D)J joining during B-cell ontogeny. Genes Dev (2013) 27:2439–44 10.1101/gad.227165.113 24240234 PMC3841733

[120] Wuerffel R Wang L Grigera F Manis J Selsing E Perlot T S-S synapsis during class switch recombination is promoted by distantly located transcriptional elements and activation-induced deaminase. Immunity (2007) 27:711–22 10.1016/j.immuni.2007.09.007 17980632 PMC4979535

[121] Xu Z Zan H Pone EJ Mai T Casali P Immunoglobulin class-switch DNA recombination: induction, targeting and beyond. Nat Rev Immunol (2012) 12:517–31 10.1038/nri3216 22728528 PMC3545482

[122] Garrett FE Emelyanov AV Sepulveda MA Flanagan P Volpi S Li F Chromatin architecture near a potential 3’ end of the *Igh* locus involves modular regulation of histone modifications during B-cell development and in vivo occupancy at CTCF sites. Mol Cell Biol (2005) 25:1511–25 10.1128/MCB.25.4.1511-1525.2005 15684400 PMC548023

[123] Thomas-Claudepierre AS Schiavo E Heyer V Fournier M Page A Robert I The cohesin complex regulates immunoglobulin class switch recombination. J Exp Med (2013) 210:2495–502 10.1084/jem.20130166 24145512 PMC3832931

[124] Enervald E Du L Visnes T Björkman A Lindgren E Wincent J A regulatory role for the cohesin loader NIPBL in nonhomologous end joining during immunoglobulin class switch recombination. J Exp Med (2013) 210:2503–13 10.1084/jem.20130168 24145515 PMC3832922

[125] Cai Y Jin J Yao T Gottschalk AJ Swanson SK Wu S YY1 functions with INO80 to active transcription. Nat Struct Mol Biol (2007) 14:872–4 10.1038/nsmb1276 17721549

[126] Wu S Shi Y Mulligan P Gay F Landry J Liu H A YY1-INO80 complex regulates genomic stability through homologous recombination-based repair. Nat Struct Mol Biol (2007) 14:1665–72 10.1038/nsmb1332 18026119 PMC2754171

[127] Zaprazna K Atchison ML YY1 controls immunoglobulin class switch recombination and nuclear activation-induced deaminase levels. Mol Cell Biol (2012) 32:1542–54 10.1128/MCB.05989-11 22290437 PMC3318595

[128] Hwang SS Kim YU Lee S Jang SW Kim MK Koh BH Transcription factor YY1 is essential for regulation of the Th2 cytokine locus and for Th2 cell differentiation. Proc Natl Acad Sci U S A (2013) 110:276–81 10.1073/pnas.1214682110 23248301 PMC3538243

[129] Yancopoulous GD Alt FW Developmentally controlled and tissue-specific expression of unrearranged V_H_ gene segments. Cell (1985) 40:271–81 10.1016/0092-8674(85)90141-2 2578321

[130] Baumeister P Luo S Skarnes WC Sui G Seto E Shi Y Endoplasmic reticulum stress induction of the Grp78/BiP promoter: activating mechanisms mediated by YY1 and its interactive chromatin modifiers. Mol Cell Biol (2005) 25:4529–40 10.1128/MCB.25.11.4529-4540.2005 15899857 PMC1140640

[131] Kassardjian A Rizkallah R Riman S Renfro SH Alexander KE Hurt MM The transcription factor YY1 is a novel substrate for Aurora B kinase at G2/M transition of the cell cycle. PLoS One (2012) 7(11):e50645 10.1371/journal.pone.0050645 23226345 PMC3511337

[132] Rizkallah R Hurt MM Regulation of the transcription factor YY1 in mitosis through phosphorylation of its DNA-binding domain. Mol Cell Biol (2009) 20:4766–76 10.1091/mbc.E09-04-0264 19793915 PMC2777106

[133] Deng Z Wan M Sui G PIASy-mediated sumoylation of Yin Yang 1 depends on their interaction but not the RING finger. Mol Cell Biol (2007) 27:3780–92 10.1128/MCB.01761-06 17353273 PMC1899983

[134] Ficzycz A Eskiw C Meyer D Marley KE Hurt M Ovseneck N Expression, activity, and subcellular localization of the Yin Yang1 transcription factor in *Xenopus* oocytes and embryos. J Biol Chem (2001) 276:22819–25 10.1074/jbc.M011188200 11294833

[135] Ficzycz A Ovsenek N The Yin Yang 1 transcription factor associates with ribonucleoprotein (mRNP) complexes in the cytoplasm of *Xenopus* oocytes. J Biol Chem (2002) 277:8382–7 10.1074/jbc.M110304200 11734562

[136] Palko L Bass HW Beyrouthy MJ Hurt MM The Yin Yang-1 (YY1) protein undergoes a DNA-replication-associated switch in localization from the cytoplasm to the nucleus at the onset of S phase. J Cell Sci (2004) 117:465–76 10.1242/jcs.00870 14702388

[137] Yue R Kang J Zhao C Hu W Tang Y Liu X Beta-arrestin1 regulates zebrafish hematopoiesis through binding to YY1 and relieving polycomb group repression. Cell (2009) 139:535–46 10.1016/j.cell.2009.08.038 19879840

[138] Krippner-Heidenreich A Walsemann G Beyrouthy MJ Speckgens S Kraft R Thole H Caspase-dependent regulation and subcellular redistribution of the transcriptional modulator YY1 during apoptosis. Mol Cell Biol (2005) 25:3704–14 10.1128/MCB.25.9.3704-3714.2005 15831475 PMC1084290

[139] Sucharov CC Helmke SM Langer SJ Perryman MB Bristow M Leinwand L The Ku protein complex interacts with YY1, is up-regulated in human heart failure, and represses alpha myosin heavy-chain gene expression. Mol Cell Biol (2004) 24:8705–15 10.1128/MCB.24.19.8705-8715.2004 15367688 PMC516749

[140] Sucharov CC Mariner P Long C Bristow M Leinwand L Yin Yang 1 is increased in human heart failure and represses the activity of the human alpha-myosin heavy chain promoter. J Biol Chem (2003) 278:31233–9 10.1074/jbc.M301917200 12754214

[141] Lai F Orom UA Cesaroni M Beringer M Taatjes DJ Blobel GA Activating RNAs associate with Mediator to enhance chromatin architecture and transcription. Nature (2013) 494:497–501 10.1038/nature11884 23417068 PMC4109059

